# Peer-led theoretically Desinged HIV/AIDS prevention intervention among students: a case of health belief model

**DOI:** 10.1186/s12889-021-12445-6

**Published:** 2022-01-05

**Authors:** Hoda Joorbonyan, Mohtasham Ghaffari, Sakineh Rakhshanderou

**Affiliations:** Present Address: Health Education & Health Promotion, School of Public Health and Safety, Shahid Beheshti University of Medical Sciences, Tabnak Ave., Daneshjou Blvd., Velenjak, P.O. Box 19835-35511, Tehran, Iran

**Keywords:** AIDS, Adolescents, Health belief model, Peer group

## Abstract

**Background & aim:**

HIV/AIDS is one of the most dangerous viruses known in the world. In addition, considering its fatality rate and high cost of care, it is a serious threat to the health and economy of social communities. Adolescents are one of the high-risk groups. One of the most effective ways to prevent this disease is to promote healthcare, raise awareness, and change health-related beliefs and attitudes. This study aims at determining the effect of peer education, based on the health belief model, on the preventative measures against AIDS adopted by girls.

**Methods & materials:**

In this empirical-interventionist study two schools were randomly selected, one of which was considered as the intervention group and the other as the control group. The classes were also randomly selected and 80 students from each school took part in the project following the entry criterion. A questionnaire with acceptable validity and reliability was used to collect data. In this study a few bright students were chosen as peer educators after being trained. The intervention group (*N* = 80) received 4 sessions of 60-min education through training, lectures, question and answer, and group discussion, But the control group received no instruction. The posttest was administered two months after the treatment. The data was fed into the SPSS 16. Finally, T-test, Chi-Square, and ANCOVA were employed to analyze the data.

**Results:**

The average scores obtained from the intervention group and the control group were not significantly different in terms of awareness level, perceived susceptibility, perceived severity, perceived benefits, perceived barriers, perceived self-efficacy, behavioral intention, and behavior in baseline (*P*>0.05). Two months after the intervention there was a significant increase in the average scores of all the variables in the experimental group (*P* > 0.05). However, there was no significant change in the scores of the control group (*P* > 0.05).

**Conclusion:**

Following a health belief model focusing on peer eduaction among high school girls, the intervention eduaction can affect awareness level, susceptibility, severity, benefits, perceived barriers, perceived self-efficacy, behavioral intention, and finally avoidance of high-risk behavior.

## Background

AIDS, one of the most dangerous viral diseases known in the world, is the result of a virus infection affecting the immunity system. In addition, considering its fatality rate and high cost of care, it is a serious threat to the health and economy of social communities [1].

For instance, 95% of all infections and 90% of all HIV / AIDS deaths occur in underdeveloped countries [2]. Still high on the agenda, HIV has claimed over 33 million lives in the world so far. According to World Health Organizaton (WHO), 690,000 lives have been lost due to AIDS-realted issues in 2020 [3]. Due to common misconceptions about AIDS, this disease typically adds some mental and emotional complications to the physical ones, totally overwhelming the patients [4]. According to existing studies, the scale of the disease is growing rapidly in the Eastern Mediterranean and the Middle East, such as Iran [5]. The statistics demonstrate that there was a 24% increase in the number of cases between 2010 and 2019. In 2019 there were 38 million sufferers, 700,000 of whom died within one year. By the end of 2019, approximately 59,000 cases had been identified in Iran, almost a quarter of whom were women of 15 years of age and beyond (16000) and about 75% were men of the same age group (43000) [6, 7].

Today, AIDS is no longer considered as a disease but as a sociocultural phenomenon and personal behavior when it targets victims of various age groups, in particular adolescents [5]. As mentioned earlier, teenagers are the most vulnerble age group. WHO estimates that on a daily basis 7000 new cases are added (5 people each minute in the 10–25 age group) [3]. Adolescence is the period of experimentation and personal choices marking the onset of sexual identity. In general, teenagers and young adults do not see themselves as susceptible to sexually transmitted diseases (STD), in particular AIDS, and often conceal their sexual experimentation [8, 9].

Cultural norms and sexual roles pose serious risks to the vulnerable youth. Female teenagers are highly prone to the disease as the statistical evidence suggets that more than a fifth of the cases, twice as many as the mature population, are young girls [9]. In Comparison to boys, girls are more likely to be infected with HIV at an early age, which is why the global prevalence of AIDS among women is twice as high as men at the same age. In addition to women’s physiological vulnerability to HIV, there are gender inequalities, including vulnerability to rape, sex with older men, and unequal access to education and economic opportunities, which exacerbate the risks of AIDS for girls and young women [10].

Lots of research has been conducted on AIDS, concluding that the most effective preventative measure is education and awareness-raising. Indeed, WHO highlights social awareness as the number one factor in preventing and curbing the spread of AIDS [11].

Peer education has been employed as an effective strategy to combat AIDS around the globe [12, 13]. Accordingly, people with shared characteristics such as ader, culture, education, and place of residence are assigned the task of imparting information with the aim of building awareness and changing attitude and behavior among individuals, groups, and communities [14]. Relying on all five senses, this method fosters thinking and creativity and promotes all-encompasssing participation in all the stages of planning, implementation, and even evaluation [12]. Studies such as Babazade et al., Sioki et al., Adeomi, and Calloway are a few of those investigating similar methods to prevent AIDS [15–18]. Various research projects confirm that the school setting can be the optimal place to access the largest number of teenagers. School is the right place to implement educational programs to counteract high-risk behavior before it is internalized in young people [19].

**Fig. 1:**
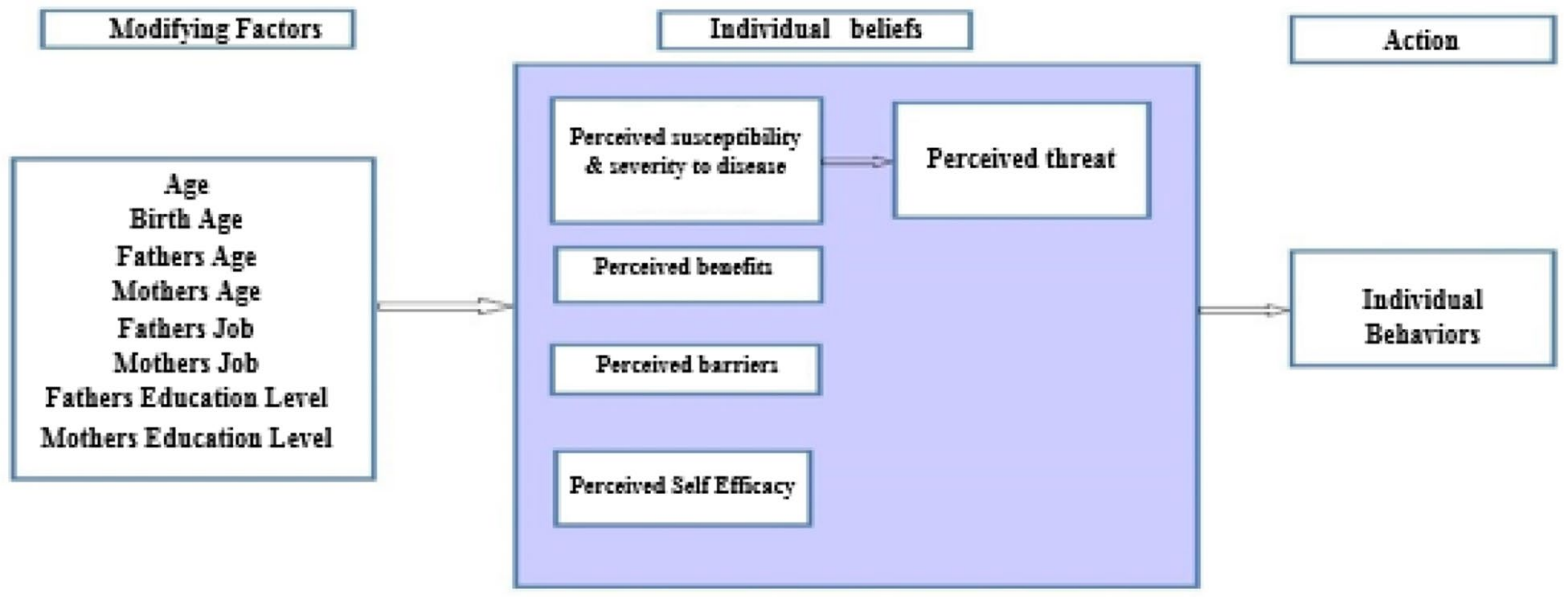
Health belief model’s components and links

Today most behaviorists underscore the fact that health interventions should be based on a model. One of the most effective and widely used models is Health Belief Model (HBM). HBM is a tool that scientists use to try and predict health behaviors. It was originally developed in the 1950s and updated in the 1980s by Rosenstock at the United States Public Health Service. The health belief model has different constructs. The percieved susceptibilty is seeing oneself vulnerable to the disease. The percieved severity is understanding the severity and the seriousness of the illness. The percieved benefits relate to understanding the benefits derived from adopting preventative measures. The perceived barriers means believing the barriers to preventative measures. The perceived self-efficacy entails having enough self-confidence to exhibit particular behavior (Fig. 1) [20].

Behaviorists see ‘intervention education on the basis of a model’, which is intended to raise awareness and strengthen beliefs, as a prelude to altering unhealthy measures and adopting preventive ones [20]. Previous interventions based on health belief model confirm the efficacy of interventions in shaking the beliefs of the research population and adopting preventive measures against AIDS [21, 22].

Peer education enhances self-confidence, self-esteem, assertiveness, self-efficacy, knowledge, and attitude, as well as upgrading the health skills of the trainers and the trainees [23]. On the other hand, the health belief model deals with the relationship between health beliefs and a person’s behavior, focusing on preventing and curbing diseases [24]. As a result, the present study aims at investigating the effects of peer education on girls’ preventive measures against AIDS following the health belief model. It is hoped that vital steps are taken to raise awareness to improve the health of teenagers as well as future mothers.

## Materials & methods

### Study design and sampling

**Fig. 2:**
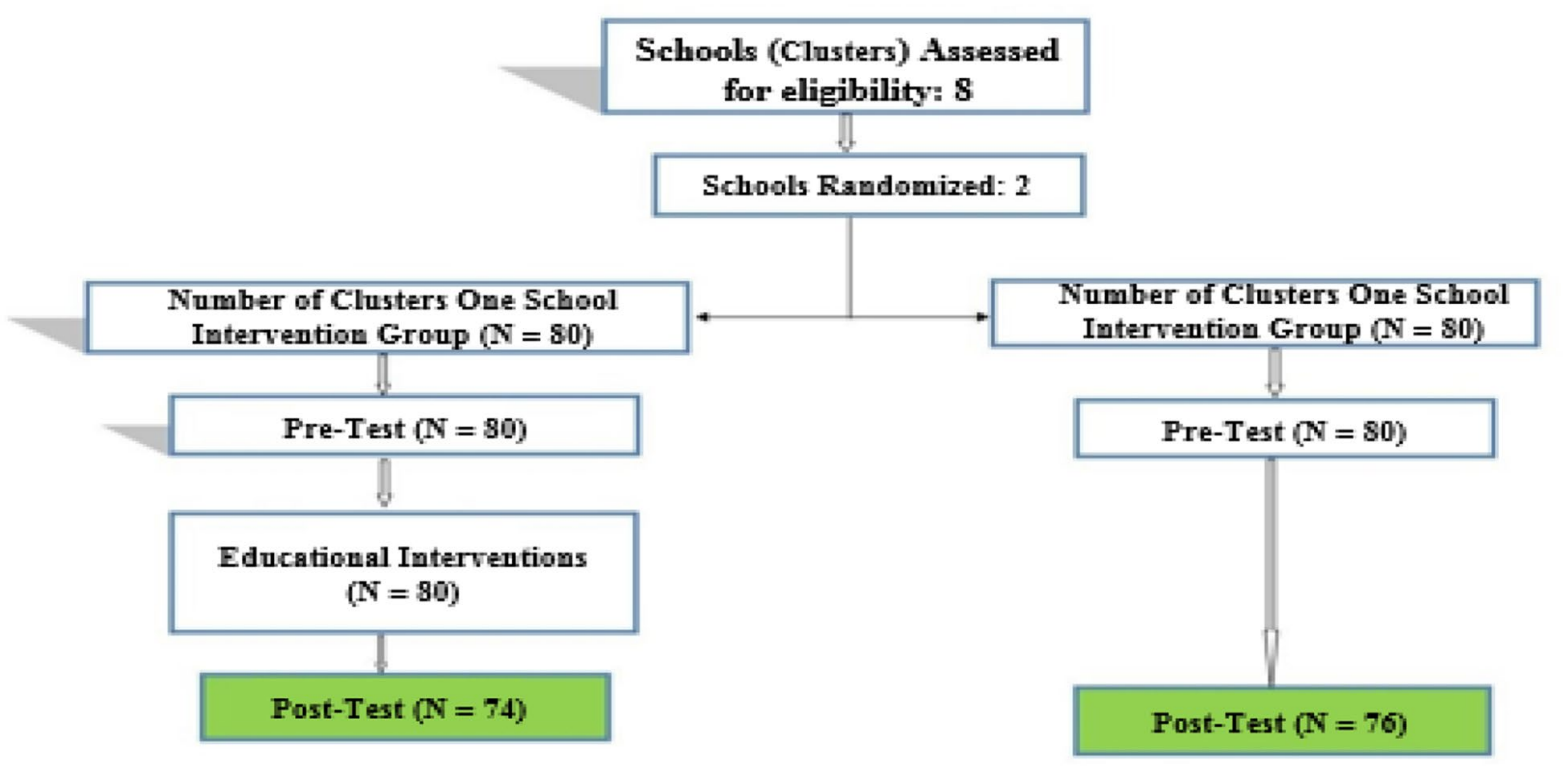
Schematic diagram of designed interventions for promotion of AIDS prevention

This empirical research with random cluster sampling was conducted in high schools in Ramsar, Iran. Two schools were randomly selected from that region. One was selected as the intervention group and the other as the control group. Classes were also randomly selected and students entered the research groups as participants. Figure 2 clearly illustrates the research process.

### Sample size

According to the research conducted in the same field [25] and using the following formula, sample size was calculated with 10% drop 80 people in each group.

$$\boldsymbol{N}=\frac{{\left[{\boldsymbol{Z}}_{\left(\mathbf{1}-\frac{\boldsymbol{\alpha}}{\mathbf{2}}\right)}+{\boldsymbol{Z}}_{\left(\mathbf{1}-\boldsymbol{\beta} \right)}\right]}^{\mathbf{2}}\left({\boldsymbol{S}}_{\mathbf{1}}^{\mathbf{2}}+{\boldsymbol{S}}_{\mathbf{2}}^{\mathbf{2}}\right)}{\Delta ^{\mathbf{2}}}=\mathbf{73}$$

$${\boldsymbol{Z}}_{\left(\mathbf{1}-\boldsymbol{\beta} \right)}=\mathbf{1.28}\kern1em {\boldsymbol{S}}_{\mathbf{1}}=\mathbf{5.08}\kern1.25em {\boldsymbol{S}}_{\mathbf{2}}=\mathbf{5.58}\kern1.25em {\mathbf{\Delta }}^{\mathbf{2}}=\mathbf{4.84}\kern1.25em {\boldsymbol{Z}}_{\left(\mathbf{1}-\frac{\boldsymbol{\alpha}}{\mathbf{2}}\right)}=\mathbf{1.96}$$

### Inclusion & exclusion criteria

The inclusion criteria for research included female students, voluntary participation, informed consent on the part of students and their parents, and studying at 10th, 11th, and 12th grades at high school (ages 15 to 18). The subjects’ reluctance to participate at any stage, more than one session absence, and leaving the school were considered as exclusion criteria.

### Measures

The data collection method, employed in the previous research, was a questionnaire who’s designed by Mohtasham Ghaffari (2007) and Validity & reliability have been determined and had these two sections:

*Part one:* Demographic questions about age, Birth rate, Fathers age, Mothers age, Fathers job, Mothers job, Fathers education level, Mothers education level. *Part two:* Constructs of the health belief model (which includes; knowledge, perceived susceptibility, perceived severity, perceived benefits, perceived barriers, perceived self-efficacy, behavioral intention, and behavior) (Table 1).

**Table 1:** Description of study instrument

Construct	No. of Items (Format)	Scoring (Range)
***1) Knowledge;*** refers to a theoretical or practical understanding of subject.	11 items (true – false - don’t know)	‘Correct’ response = 2, ‘don’t know ‘response = 1, ‘incorrect’ response = 0 (0–22)
***2) Perceived Susceptibility;*** refers to subjective assessment of risk of developing a health problem.	6 items / 5-point Likert Scale (strongly disagree to strongly agree)	strongly disagree = 1, disagree = 2, no idea = 3, agree = 4, strongly agree = 5 (6–30)
***3) Perceived Severity;*** refers to the subjective assessment of severity of a health problem and its potential consequences.	4 items / 5-point Likert Scale (strongly disagree to strongly agree)	strongly disagree = 1, disagree = 2, no idea = 3, agree = 4, strongly agree = 5 (4–20)
***4) Perceived Benefits;*** refers to an individual’s perception of the positive aspects of health measures.	15 items / 5-point Likert Scale (strongly disagree to strongly agree)	strongly disagree = 1, disagree = 2, no idea = 3, agree = 4, strongly agree = 5 (15–75)
***5) Perceived Barriers;*** refers to an individual’s perception of the psychological and financial costs of health measures.	19 items (yes - to some extent - no)	‘yes’ response = 1, ‘to some extent response = 2, ‘No’ response = 3 (19–57)
***6) Perceived Self-efficacy;*** refers to an individual’s perception of his or her competence to successfully perform a behavior	8 items / 5 point Likert Scale (strongly disagree to strongly agree)	strongly disagree = 1, disagree = 2, no idea = 3, agree = 4, strongly agree = 5 (8–40)
***7) Behavioral Intention;*** refers to a person’s perceived probability or “subjective probability” that he or she will engage in a given behavior.	9 items / 5-point Likert Scale (strongly disagree to strongly agree)	strongly disagree = 1, disagree = 2, no idea = 3, agree = 4, strongly agree = 5 (9–45)
*8) Behavior;* refers preventative behaviors associated with HIV/AIDS	8 items / 5-point Likert Scale (always to never)	Always = 5, often = 4, sometimes = 3, rarely = 2, never = 1 (8–40)

To determine the validity of the questionnaire – face validity and content validity – an expert panel of 15 researchers specializing in health sciences, behavior sciences, and social sciences were employed. All the eminent researchers had relevant academic experience in the area of adolescence and AIDS [25]. The reliability of the questionnaire was re-evaluated in this study. To determine the reliability of the Knowledge questionnaire test-retest method with a 15-day interval was used. In addition, to determine the reliability of the scales of perceived susceptibility, perceived severity, perceived benefits, perceived self-efficacy, and behavioral intentions, Cronbach’s alpha internal consistency method with a sample of 160 was utilized. The reported reliability coefficient for Knowledge was r = 0.86, scales of perceived susceptibility α = 0.78, perceived severity α =0.70, perceived benefits α =0.83, perceived barriers α =0.82, perceived self-efficacy α =0.77, and behavioral intentions α =0.78.

### Intervention

After the pretest was administered, the educational needs were determined and the educational content was designed, making use of the reliable and principal sources authorized by Ministry of Health as well as considering what students need to know regarding preventive measures against AIDS. Then enthusiastic students with high academic, social, and training skills prepared to cooperate were chosen to form the 4-member intervention team of peer educators. The peer educators underwent four 60-min sessions by the researcher. During these sessions peer educators were trained about the educational objectives and the educational materials on AIDS on the basis of the constructs of health belief model. Then four educational sessions were held by the peer educators for their peers. Training was through lectures and group discussions. Facts and figures associated with the rate of AIDS were employed for the purpose of the perceived susceptibility. Images and pictures of AIDS sufferers were used for the perceived severity. In addition, for the purpose of perceived barriers educational materials were adopted in a way that individuals compare and contrast optimal behavior costs against the AIDS-related ones. The materials in relation to perceived benefits included awareness-raising and preventive measures against AIDS (self-control, personal hygiene, AIDS test, discussion with parents over high-risk behavior and AIDS, rejection or postponement of perilous proposals). When it came to perceived self-efficacy, educational materials were designed so as to raise individual’s perception of one’s ability to adopt adequate health measures. The control group received no instruction. Two months after the educational intervention, the questionnaires were completed again by both groups. The extent of the effect of educational intervention was measured.

### Ethical considerations

At first, a permission was obtained from the university to conduct the study and attend the schools. The participants were allowed to enter and leave the study at any time. Suitable conditions were provided for a proper understanding of questions and responses for the subjects. Written informed consent was obtained from the study participants and from the parents of minor participants. The study on which these data analyses are based was approved by the Ethical Board Committee of Shahid Beheshti University of Medical Sciences (IR.SBMU.PHNS.REC.1398.152).

After the completion of the intervention, the control group was also trained through the slides used in the experimental group. All methods were performed in accordance with the relevant guidelines and regulations.

### Procedure

The samples were assured about the confidentiality of their specifications and information. They were also told that, their information will only be used for the purpose of this study and the data collection. Coding was used to ensure the confidentiality of information.

### Data analysis

To check the normality of the data, Kolmogorov-Smirnov test was used. Paired t-test was employed to compare the scores of awareness, susceptibility, severity, benefits, barriers, self-efficacy, and behavioral intentions in each group both before the intervention and two months after the training. Analysis of Covariance (ANCOVA) was utilized to compare the mean scores between the groups. The level of statistical significance was set to be lower than 0.05. Data were analyzed by SPSS 16 software.

## Results

The sample size was 80 for each group. After the intervention 6 samples were lost in the intervention group and 4 samples in the control group for various reasons. Data analysis was performed with this size of samples.

The questionnaire was completed in both groups in a complete and precise manner. Homogenization was done in the two groups by controlling variables such as age, birth rate, parent’s age, parent’s job, parent’s level of education. The results showed no significant difference in terms of these variables (*P* > 0.05), (Table 2).

**Table 2:** Demographic and background variables in intervention and control groups before the intervention

Variable	Subgroup	Intervention group (*N* = 74) N (%)	Control group (*N* = 76) N (%)	*P* –value*
Age	15	14 (18.9)	10 (13.2)	0.707
	16	21 (47.3)	23 (43.4)	
	17	22 (77)	21 (71.1)	
	18	17 (100)	22 (100)	
Birth rate	1	37 (50)	33 (43.4)	0.367
	2	25 (83.8)	34 (88.2)	
	3	12 (100)	9 (100)	
Fathers age	35–45	29 (40.3)	29 (44.6)	0.608
	Upper of 45	43 (100)	36 (100)	
Mothers age	30–40	33 (44.6)	31 (43.1)	0.851
	Upper of 40	41 (100)	41 (100)	
Fathers job	Unemployed	5 (6.8)	14 (18.4)	0.077
	Manual worker	4 (12.2)	8 (28.9)	
	Employee	12 (28.4)	12 (44.7)	
	Freelance job	53 (100)	42 (100)	
Mothers job	Housewife	62 (83.8)	58 (76.3)	0.253
	Manual worker	12 (100)	18 (100)	
Fathers education level	None and Primary	18 (24.7)	29 (40.8)	0.112
	Secondary or higher	24 (57.5)	17 (64.8)	
	Highest education	31 (100)	25 (100)	
Mothers education level	None and Primary	15 (20.3)	21 (28)	0.163
	Secondary or higher	22 (50)	28 (65.3)	
	Highest education	37 (100)	26 (100)	

The results revealed that the intervention was successful in improving constructs of the HBM significantly in participants (Table 3).

**Table 3:** Comparison of groups in terms of HBM constructs before and after intervention

Constructs	Groups	Before intervention Mean ± SD	After intervention Mean ± SD	Mean Difference	*P* value*
Knowledge	Intervention	75.58 ± 14.34	90.2 ± 13.6	14.62 ± 0.74	0.000
	Control	66.7 ± 16.28	68.86 ± 15.17	2.16 ± 1.11	
Perceived Susceptibility	Intervention	15.51 ± 3.14	19.58 ± 3.13	4.07 ± 0.01	0.000
	Control	15.4 ± 3.25	16.01 ± 2.99	0.61 ± 0.26	
Perceived Severity	Intervention	11.6 ± 2.20	13.55 ± 2.47	1.95 ± 0.27	0.000
	Control	12.09 ± 2.65	12.03 ± 2.52	−0.06 ± 0.13	
Perceived Benefits	Intervention	57.32 ± 8.48	65.25 ± 5.9	7.93 ± 2.58	0.000
	Control	53.47 ± 8.57	54.5 ± 8.31	1.03 ± 0.26	
Perceived Barriers	Intervention	46.82 ± 5.7	49.17 ± 5.88	2.35 ± 0.18	0.001
	Control	45.01 ± 6.72	45.4 ± 6.88	0.39 ± 0.16	
Perceived Self- Efficacy	Intervention	32.72 ± 4.74	34.28 ± 4.77	1.56 ± 0.03	0.002
	Control	31.64 ± 5.18	31.65 ± 5.12	0.01 ± 0.06	
Behavioral Intention	Intervention	35.54 ± 5.06	38.06 ± 5.05	2.52 ± 0.01	0.000
	Control	33.4 ± 5.5	33.86 ± 5.35	0.46 ± 0.15	
Behavior	Intervention	30.71 ± 5.71	32.7 ± 5.8	1.99 ± 0.09	0.003
	Control	29.06 ± 6.89	29.56 ± 6.72	0.5 ± 0.17	

*Analysis of Covariance (ANCOVA)

*SD* Standard Deviation

**Fig. 3:**
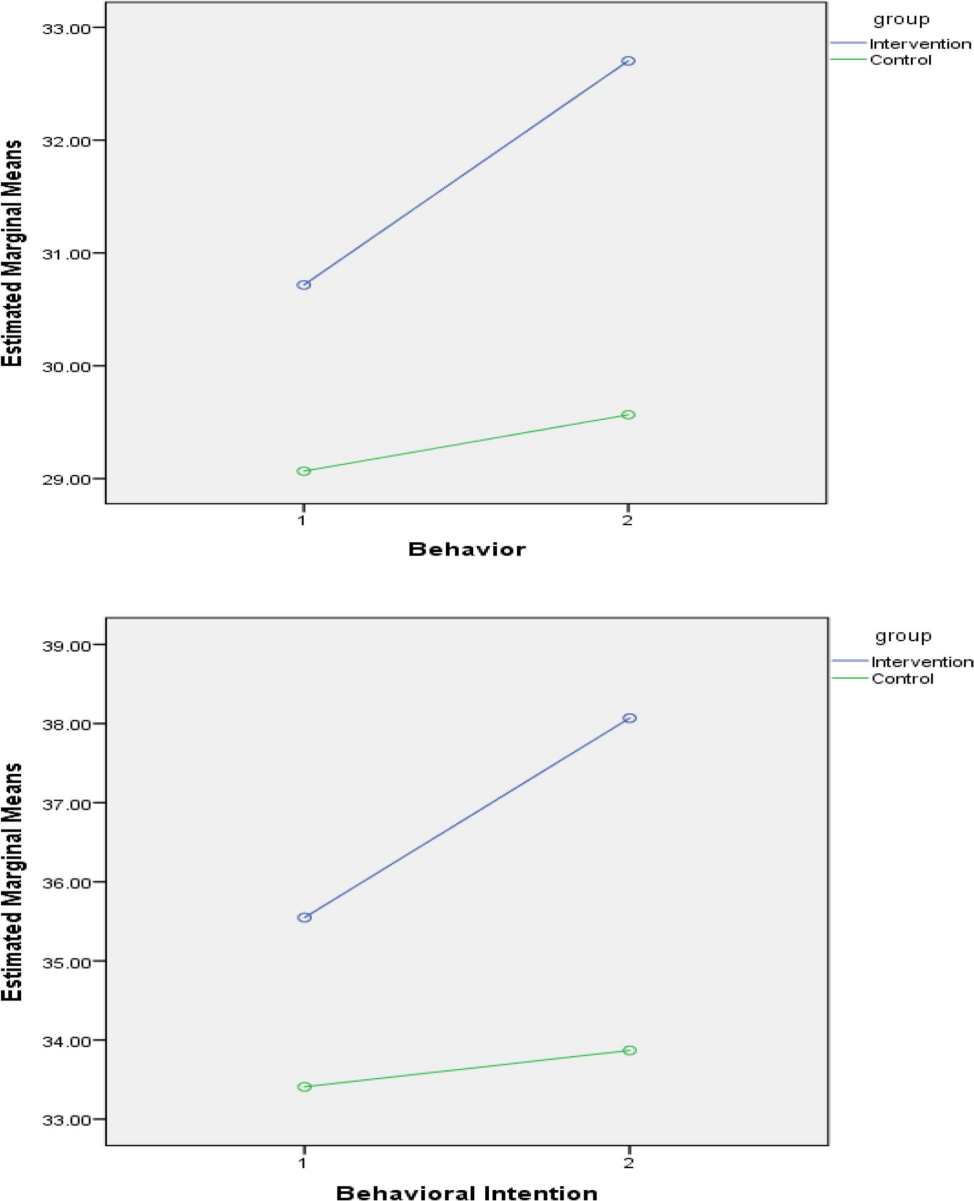
Estimated Marginal Means of Behavioral Intention and Behavior

The mean score of behavioral intention and behavior in the experimental and control groups before and after the intervention is presented in Fig. 3.

## Discussion

The chief purpose of this research is to determine the effect of peer education on the female teenagers’ preventive measures against AIDS based on health belief model. The review of literature confirms the efficacy of peer education in preventing this disease [12, 13, 16, 18, 26]. The findings of this study show that there is a significant difference between the intervention group and the control group with respect to the average scores of knowledge after the educational intervention. These results are consistent with the findings of studies following the health belief model [1, 25, 27–30]. The significant difference in awareness scores in the intervention group results from the effect of educational intervention on students’ enhanced awareness. However, such a significant difference was not observed in the control group.

Most interventions based on theories and models of behavior change have been commonly used in interventions aiming to improve HIV-related knowledge, as it has been found that education interventions on HIV are associated with a greater likelihood of subsequent adoption of preventive behaviors when implemented in combination with behavior change elements [31]. Faust and Yaya in a systematic review and meta-analysis reporting that 10 studies have indicated that The peer education interventions were generally found to be effective at improving HIV-related knowledge in the target population [32].

Moreover, the average score for the perceived susceptibility witnessed a significant difference for the intervention group, which is compatible with the results of several studies [25, 28, 29, 33]. According to the health belief model, a growing perception of susceptibility to a medical condition can encourage people to adopt preventive health approaches. The significant increase in the construct of perceived susceptibility is indicative of the positive effect of the educational intervention within the health belief model and peer education framework, which has made girls find themselves more susceptible to AIDS. This finding is, however, in conflict with what Pirzade & Sharifirad and Kharazi & Peyman came across in their studies [1, 27]. The former finds the reason for a slight increase in the perceived susceptibility of the experimental group in conducting research on girls and lower incidence of high-risk behavior – such as sexual experimentation, drug abuse and long-term intervention programs to sensitize girls [1]. Also the average score for the perceived severity showed a significant difference between the intervention group and the control group after the treatment. The results of other studies such as Pirzade & Sharifirad, Calloway, Ghaffari, et al., Kharazi & Peyman, Soltani, et al., Khani, et al. support the results of the current study [1, 18, 25, 27–29]. The results of this study did not match up with the findings of Ghaffari et al. [25]. This inconsistency might be put down to the difference in educational approaches and research population. What it highlights is that female students not only see themselves as prone to the disease but they also regard AIDS as a deadly disease. The health belief model can be used to accentuate the fatal consequences of this virus, leading students to adopt preventive measures against it. Furthermore, peer education is seen to enhance the extent of perceived severity in the intervention group. The results indicate that even though both groups were wanting in their perception of benefits, educational intervention could enhance the perceived benefits in the experimental group. Therefore, peer education in the form of question and answer and group discussion has been able to elucidate the benefits of adopting preventive measures against AIDS for students. This finding corroborates the results achieved by other scholars who attributed the enhanced perception of benefit to educational interventions [1, 27–30, 34]. Numerous research studies have reported on the strong correlation between the perceived benefits and the adoption of preventive measures [35]. The present study revealed that after the treatment there was a significant difference in the average score of the perceived barrier, with no difference in the control group. Therefore, after the educational intervention within the peer education framework, the experimental group saw fewer barriers to adopting preventive measures against AIDS. It seems that enhanced awareness, change of false beliefs, and group discussions led by peers can decrease the perceived barriers. It can be concluded from this study that high perceived benefits along with low perceived barriers contribute to healthy behavior and personal hygiene. The results of Pirzade & Sharifirad and Kharazi & Peyman studies [1, 27] confirm the findings of the present study, emphasizing the positive effect of educational intervention on the reduction of the perceived barriers. However, Ghafari et al. study [25] generated conflicting results. The perceived self-efficacy is a prelude to behavior. Therefore, a particular attention should be given to enhancing self-efficacy [36]. The present research showed a significant difference in the average score of the construct of self-efficacy between the two groups after the intervention. The results of other research studies – on the basis of the health belief model with students as participants – demonstrate that educational interventions contribute to enhanced perceived self-efficacy with respect to preventive measures against AIDS (27-29). Calloway considers self-efficacy as a protective factor for adolescents as well as one of the strongest predictors of behavior among all the constructs of health belief model (HBM) [18]. In this study there was a significant difference in the average score of the students’ behavioral intentions for the intervention group after the treatment. Other projects in this area came to similar results: Babazade et al., and Ghaffari et al. [15, 25, 37]. Parrot et al. indicate that positive and negative messages contribute to an increase in students’ behavioral intentions toward physical activity [38]. Calloway concluded that after the intervention people were more willing to use condoms during their sexual intercourse and a larger number of the participants inquired about AIDS test of their partners [18]. In addition, students’ behavior in the experimental group saw an improvement after the intervention when compared to the control group. The findings of Alizade et al., Taghdisi et al., and Lotfi et al. are all compatible with the results of this study [16, 34, 39]. Frank Vandermes contends that peer education contributes to a considerable improvement in children’s knowledge and behavior, which supports the arguments of this study [40].

### Limitations

The limitations of the study which can affect the generalizability of the findings include short-term intervention, impossibility of assessing the long-term effect of the intervention, individual’s responses to the questions, and inclusion of only one gender in the study.

## Conclusion

The results of the study show that educational intervention based on the health belief model within peer education framework can contribute to enhanced awareness, susceptibility, severity, benefits, perceived self-efficacy, behavioral intentions and eventually prevent high-risk behavior among female high school students. Applying this study to a similar population may lead to the prevention of AIDS in any community. Therefore, implementing healthcare policies in schools should be prioritized by authorities and public health experts. In addition, AIDS-focused education should be continuously offered to all high school students so that the required information and awareness is imparted to students with a view to changing people’s beliefs and attitudes. The results of this research project can be used in theoretical intervention strategies so as to effect health measures changes.

## Data Availability

The datasets used and analyzed during the current study are available from the corresponding author on reasonable request.

## Abbreviations

AIDS: Acquired Immune Deficiency Syndrome
HBM: Health Belief Model
